# HRMS Characterization and Antioxidant Evaluation of Costa Rican Spent Coffee Grounds as a Source of Bioactive Polyphenolic Extracts

**DOI:** 10.3390/foods14030448

**Published:** 2025-01-30

**Authors:** Mirtha Navarro-Hoyos, Luis Felipe Vargas-Huertas, Juan Diego Chacón-Vargas, Valeria Leandro-Aguilar, Diego Alvarado-Corella, Jose Roberto Vega-Baudrit, Luis Guillermo Romero-Esquivel, Andrés Sánchez-Kopper, Andrea Monge-Navarro, Andrea Mariela Araya-Sibaja

**Affiliations:** 1 BIODESS, Department of Chemistry, University of Costa Rica, San Pedro de Montes de Oca 11501, Costa Rica; luis.vargashuertas@ucr.ac.cr (L.F.V.-H.); juan.chaconvargas@ucr.ac.cr (J.D.C.-V.); valeria.leandroaguilar@ucr.ac.cr (V.L.-A.); luis.alvaradocorella@ucr.ac.cr (D.A.-C.); tpc@codeti.org (A.M.-N.); 2 I&D+i Department, INNOBIOTIQ, Pavas 10107, Costa Rica; aaraya@cenat.ac.cr; 3 Chemistry Department, Georgetown University, Washington, DC 20057, USA; 4 National Laboratory of Nanotechnology, CENAT, Pavas 10109, Costa Rica; jvegab@gmail.com; 5 Costa Rica Institute of Technology, Department of Chemistry, Environmental Protection Research Center, Cartago 30109, Costa Rica; lromero@itcr.ac.cr; 6 Department of Chemistry, Chemistry and Microbiology Research and Services Center, Costa Rica Institute of Technology, Cartago 30109, Costa Rica; ansanchez@itcr.ac.cr; 7 National Technical University, Central Campus, Alajuela 20101, Costa Rica

**Keywords:** spent coffee grounds, waste, polyphenols, caffeoylquinic acids, feruloylquinic acids, atractylegenins, antioxidant activity

## Abstract

Spent coffee grounds constitute a waste product that has attracted potential interest as a rich source of secondary metabolites such as polyphenolic compounds with antioxidant properties. In this work, aqueous extracts from samples of different spent coffee grounds from Costa Rica were prepared and analyzed using ultra-performance liquid chromatography coupled with high-resolution mass spectrometry using a quadrupole time-of-flight analyzer (UPLC-QTOF-ESI MS). This allowed for the identification of twenty-one compounds, including fourteen phenolic acids, three caffeoylquinic lactones, and four atractyligenin diterpenes. In addition, using UPLC coupled with a diode array detector (UPLC-DAD), we quantified the levels of caffeine (0.55–3.42 mg/g dry weight [DW]) and six caffeoylquinic and feruloylquinic acids (0.47–5.34 mg/g DW). The highest value was found for the fine-grind sample (EXP), both for phenolic acids and for total polyphenols (9.59 mg gallic acid equivalents [GAE]/g DW), compared to 2.13 and 1.70 mg GAE/g DW for the medium-grind (GR) and coarse-grind samples (PCR), respectively. The results obtained from the antioxidant evaluations using the 2,2-diphenyl-1-picrylhydrazyl assay (IC_50_ 0.0964–6.005 g DW/L), the ferric-reducing antioxidant power (PFRAP) analysis (0.0215–0.1385 mmol FeSO_4_/g DW), the oxygen radical absorbance capacity (ORAC) assessment (45.7–309.7 μmol Trolox/g DW), and the Trolox equivalent antioxidant capacity (TEAC) assay (3.94–23.47 mg Trolox/g DW) also showed the best values for the fine-grind sample, with results similar to or higher than those reported in the literature. Statistical Pearson correlation analysis (*p* < 0.05) indicated a high correlation (R ≥ 0.842) between all antioxidant analyses, the total polyphenols, and the phenolic acid quantification using UPLC-DAD. These results show the potential for further studies aiming to exploit this waste product’s bioactive properties, constituting the first detailed study of spent coffee grounds from Costa Rica.

## 1. Introduction

Coffee is one of the most consumed beverages in the world and is the second-most traded commodity after petroleum, which highlights its immense global market share [1]. It has been estimated that roughly 2.25 billion cups of coffee are served every day and that more than 80% of the world’s population consumes at least one caffeine-containing beverage on a daily basis [2].

The growth of coffee consumption at the global level has in turn increased the volume of spent coffee grounds (SCGs) generated as waste, therefore creating a challenge for waste management [3]. A diversity of constituents including chlorogenic acids, pyrazines, furans, alkaloids, melanoidins, and others are responsible for coffee’s organoleptic characteristics [4].

Despite being a waste material, SCGs’ chemical composition reveals them to be a rich source of antioxidants and other high-value compounds [5]. SCGs have been identified as a source of organic compounds such as fatty acids, lignin, cellulose, hemicellulose, and other polysaccharides [6]. SCGs have also been explored for applications such as the production of bioethanol [7], biodiesel [8], bio-adsorbents for water treatment [9,10], and extracts of bioactive molecules such as polyphenols and caffeine [11,12].

In addition, the extraction of antioxidants from coffee waste has been applied in cosmetic products and several food products and formulations as either a preservation treatment or an enrichment step [13]. In the food industry in particular, considering the adverse effects of synthetic antioxidants at high concentrations and their low thermal stability in heat processing, there is a growing trend of substituting synthetic antioxidants with natural ones [14].

Chlorogenic acids (CGAs) are the main phenolic compounds in coffee, formed by the esterification of quinic acid with hydroxycinnamic acids, such as caffeic, ferulic, and p-coumaric acids [15,16]. Their metabolism follows four main pathways: direct absorption, absorption with or without hydrolysis followed by conjugation or further metabolism, microbial catabolite absorption without alteration, and microbial catabolite absorption with subsequent mammalian phase II metabolism [17,18].

Among the health benefits attributed to CGAs, their antioxidant capacity has been widely studied. After donating hydrogen atoms, CGAs are oxidized to their respective phenoxyl radicals, which are quickly stabilized through resonance. Evidence also indicates that CGAs exhibit anti-inflammatory activity by downregulating pro-inflammatory cytokines through the modulation of key transcription factors [19].

In vivo experimentation with animals has demonstrated that caffeoylquinic acid isomers (CQAs), one subgroup of CGAs, are capable of decreasing various plasma and liver lipids and improving glucose tolerance [20]. CQAs have also been evaluated for their capacity to decrease blood pressure, including the stimulation of NO production through the endothelial-dependent pathway, the reduction of free radicals through inhibiting NAD(P)H oxidase expression and activity, and the inhibition of angiotensin-converting enzyme [21].

Moreover, CQAs have been identified as a potential treatment for cancer and were approved by the China Food and Drug Administration for phase I and II clinical trials in glioma patients, which showed that CQAs function as a safe differentiation inducer for solid tumors [22].

Other important but less explored CGAs present in coffee are feruloylquinic acid isomers (FQAs), which have been reported to exhibit anti-inflammatory effects by inhibiting LPS-induced NO production and IL-1β, IL-6, iNOS, COX-2, and NF-κB expression in a dose-dependent manner in RAW 264.7 cells [23]. Another study on the isomers of both CQAs and FQAs also described them as potent antioxidants with a greater capacity to scavenge hydroperoxyl radicals in polar and lipidic media than other potential antioxidants [24].

In turn, coumaroylquinic acid isomers (CoQAs) have been identified as potential astringent compounds [25] and also as compounds with antioxidant activity when evaluating their hydroxyl radical-scavenging capacity [26]. A study dealing with the effect of in vitro gastrointestinal digestion on the bio-accessibility of polyphenols showed that 3-CoQA was easily released from food matrices after incubation with digestive fluids, contributing to the antiproliferative activity against two models of human colon adenocarcinoma cell lines [27].

On the other hand, atractyloside I, a diterpenoid glycoside that has been reported in coffee beans [28], has been found to block ANT2 expression, promoting the activation of adenosylate-activated protein kinase (AMPK), decreasing mTOR activity, and promoting autophagy activation, thus accelerating the degradation of accumulated lipids in the liver induced by high-fat-content diets [29].

In addition, treatment with atractyloside has been reported to reduce blood glucose, possibly as a consequence of restored genes and pathways, providing evidence for a new therapy for type 2 diabetes [30]. In turn, atractyligenin, the aglycone isolated from coffee silverskin, has been shown to exhibit anti-photoaging effects by recovering the altered fibroblast morphology induced by UV light and suppressing UVA-mediated matrix metalloprotease generation via the MAPK and AP-1 signaling pathways [31].

Hence, the objective of this work was to obtain extracts of the most common types of Costa Rican SCGs in order to characterize their main secondary metabolites through ultra-performance liquid chromatography coupled with high-resolution mass spectrometry using a quadrupole time-of-flight analyzer (UPLC-QTOF-ESI MS) and to determine their content using UPLC coupled with a diode array detector (UPLC-DAD). In addition, this study aimed to evaluate antioxidant activity using the 2,2-diphenyl-1-picrylhydrazyl (DPPH) assay, the potassium ferricyanide-reducing antioxidant power (PFRAP) assay, the oxygen radical absorbance capacity (ORAC) assay, and the Trolox equivalent antioxidant capacity (TEAC) assay methods, applying a correlation analysis to the data obtained. This assessment aimed to contribute to the potential application of this waste product based on its bioactive properties. To the best of our knowledge, this is the first detailed study of SCGs from Costa Rica.

## 2. Materials and Methodology

### 2.1. Reagents and Solvents

Solvents of ACS or HPLC grade, for instance, methanol and acetonitrile, were acquired from Baker (Center Valley, PA, USA). Reagents such as gallic acid, DPPH, Folin–Ciocalteu reagent, sodium carbonate, 2,2′-azinobis (3-ethylbenzothiazoline-6-sulphonate) diammonium salt (ABTS), potassium ferricyanide, iron (III) chloride, and 2,2′-azobis(2-amidinopropane) dihydrochloride (AAPH) were obtained from Sigma-Aldrich (St. Louis, MO, USA).

### 2.2. Sample Preparation

The spent coffee samples corresponded to the three main types of coffee grind from the most consumed brand in Costa Rica: fine-grind espresso (EXP) coffee, with a mean particle diameter (MPD) of 0.42 ± 0.01 mm; medium-grind (GR) coffee, with an MPD of 0.58 ± 0.01 mm; and coarse-grind percolator (PRC) coffee, with an MPD of 1.18 ± 0.02 mm. For each of the three samples, 25 g was extracted with 200 mL of boiling water using a coffee maker (Oster, Coahuila, Mexico) and a new cotton fabric filter (RCR Ruteo, Cartago, Costa Rica). The extraction was repeated once, and the 400 mL of liquid was concentrated to 25 mL in a Rotavapor^®^ R-100 with a vacuum pump V-100 rotary evaporator (Buchi, DE, USA) at 40 °C and 35 mmHg. The humidity in the spent coffee samples was measured by drying 3 g of the wet material in an oven at 50 °C until a constant mass was achieved.

### 2.3. UPLC-QTOF-ESI MS Analysis

The UPLC high-resolution mass spectrometry (UPLC-HRMS) system used to analyze the composition of spent coffee extracts consisted of a Xevo G2-XS Q-TOF (Waters, Wilmslow, UK) coupled with an AQUITY H Class UPLC system with a quaternary pump. The ESI source parameters were set to a capillary voltage of 2 kV, sampling cone of 20 eV, source temperature of 150 °C, and source offset of 10 °C. The desolvation temperature was set at 450 °C, the cone gas flow at 10 L/h, and the desolvation gas flow at 900 L/h.

Measurements were performed in the MSe high-resolution negative mode using an acquisition mass range from 100 *m*/*z* to 2000 *m*/*z* and a scan rate of 0.5 s, while fragmentation was carried out using independent data acquisition with a collision energy ramp with 20 V to 30 V stored in the high-energy function. Instrument calibration was performed in the mass range of the measurements with sodium formate. Lock mass correction was applied directly to the measurements using a leucine enkephalin infusion measured every 30 s during the run. The data were analyzed using MassLynx V4.2 software from Waters (Wilmslow, UK) and mzmine 3 from mzio.

Separation was carried out on a Luna RP-C18 column (150 mm × 4.6 mm i.d. × 4 μm, Phenomenex, Torrance, CA, USA). The solvents used in the mobile phase were water with 0.1% formic acid (A) and acetonitrile (B). In this step, 5 μL of the sample was injected with a flow rate of 0.5 mL/min at 30 °C. The chromatographic gradient started at 5% B, changing to 10% B at 12 min and holding it for 22 min, then changing to 30% B at 40 min, and finally changing to 50% B at 45 min and holding it for 5 min.

### 2.4. Total Phenolics

The determination of the total phenolics was performed using a modified Singleton and Rossi method, employing the Folin–Ciocalteu (FC) reagent, which is composed of a mixture of phosphotungstic and phosphomolybdic acids. As previously reported [32], the assay comprised mixing 10 mL of Na_2_CO_3_ (7.5%) and 0.5 mL of the FC reagent with 0.5 mL of the respective extract. Subsequently, the volume was completed to 25 mL with water. A blank was prepared following the same procedure using 0.5 mL of H_2_O in place of the extract. Both the extract mixtures and the blank were kept in the dark for 1 h, and afterwards the absorbance was measured at 750 nm. The absorbance measurements obtained were extrapolated in a gallic acid calibration curve to obtain the FC-reducing capacity results, further expressed as the mg of gallic acid equivalents (GAE)/g of the extract. Each determination was performed in triplicate.

### 2.5. UPLC-DAD Analysis

For quantification purposes, measurements were performed using a Thermo PDA photo DAD coupled with a Thermo UltiMate U3000 UPLC system and a simple quadrupole MSQ Plus instrument (Thermo Fisher Scientific, San Jose, CA, USA). A Luna RP-C18 column (150 mm × 4.6 mm i.d. × 4 μm, Phenomenex, Torrance, CA, USA) with a pre-column filter (Phenomenex, Torrance, CA, USA) was used. Mobile phases A and B consisted of a combination of 0.1% formic acid in water (*v*/*v*) and 0.1% formic acid in acetonitrile (*v*/*v*), respectively. The gradient started at 5% B and increased to 8% B at 8 min, then to 9% B at 25 min, 30% B at 40 min, and finally 50% B at 45 min, holding it for 5 min. The PDA acquisition was set at 275 nm for caffeine and 325 nm for phenol acids. Calibration curves of caffeine (10–1000 ppm) and CGA (5–150 ppm) were prepared. In this step, 20 μL of the standard or sample was injected. The results were expressed as the mg of caffeine equivalents or mg of CGA equivalents per gram of the extract.

### 2.6. Antioxidant Activity Evaluation

#### 2.6.1. DPPH Analysis

A DPPH evaluation of the samples was conducted according to previously reported methods [33]. In brief, a solution of DPPH (0.25 mM) was set in ethanol. A volume of 0.5 mL of this solution was mixed with 1 mL of the sample under evaluation at different concentrations and incubated at 25 °C in the dark for 30 min. The DPPH absorbance was measured at 517 nm. Blanks were prepared for each concentration, and the DPPH absorbance was measured at 517 nm. The inhibition percentage was determined as follows:

(1) $$Inhibition\;percentage\;(\%)=\frac{\left({Abs}_{blank}-{Abs}_{sample}\right)}{{Abs}_{blank}}*100$$

The percentage of the radical-scavenging activity of the sample was plotted against its concentration to calculate IC_50_, which corresponded to the amount of the sample necessary to reach 50% radical-scavenging activity. Each sample was analyzed in three independent assays.

#### 2.6.2. PFRAP Analysis

The PFRAP assay was conducted following a modified version of the protocol described by Işil Berker et al. [34]. In brief, an aliquot of 1 mL of the sample at a correct dilution was mixed with 0.2 mL of 1 mol/L HCl, 1.5 mL of 1% potassium ferricyanide, 0.5 mL of sodium dodecyl sulfate, and 0.5 mL of 0.1% iron (III) chloride. The volume was completed to 10 mL with distilled water and incubated at room temperature for 30 min prior to measuring the absorbance at 750 nm against a blank. Calibration curves of Trolox (50–600 μmol/L) and FeSO_4_ (35–1000 μmol/L) were made. The results were expressed as the μmol Trolox equivalent or μmol FeSO_4_ equivalent per gram of the extract.

#### 2.6.3. TEAC Analysis

For the TEAC analysis, Thaipong et al. [35]’s method was followed. In brief, an equal volume of 7.4 mmol/L ABTS (2,2′-azinobis (3-ethylbenzothiazoline-6-sulphonate) diammonium salt) and 2.6 mmol/L potassium persulfate were mixed 12 h prior to analysis to form a solution of the radical cation ABTS*^+^. Before the analysis, an aliquot of this solution was diluted with distilled water to obtain a working solution with an absorbance of approximately 1.1 mAU at 734 nm. A volume of 250 μL of the sample was mixed with 4750 μL of the working solution of ABTS*^+^ and incubated at room temperature in the dark for 2 h. The absorbance was measured at 734 nm against a blank. The inhibition percentage was calculated, and the result was interpolated in a calibration curve for Trolox (5–150 mg/L). The results were expressed as the mg Trolox equivalent (TE) per gram of the extract.

#### 2.6.4. ORAC Analysis

The ORAC analysis used to determine the antioxidant activity followed a previously described method [32,36] using fluorescein as a fluorescence probe. The reaction was performed in a 75 mM phosphate buffer (pH 7.4) at 37 °C. The final assay mixture consisted of AAPH (12 mM), fluorescein (70 nM), and either Trolox (1–8 μM) or the extract at different concentrations. The fluorescence was recorded every minute for 98 min in black 96-well untreated microplates using a Fluoroskan Ascent plate reader (ThermoFisher Scientific, San Jose, CA, USA) with 485-P excitation and 520-P emission filters. Ascent Software version 2.6 (Thermo Labsystems, Helsinki, Finland) was used to measure the fluorescence. Fluorescein was diluted from a stock solution (1.17 mM) in a 75 mM phosphate buffer (pH 7.4), while the AAPH and Trolox solutions were freshly prepared. All the reaction mixtures were prepared in duplicate, and three independent runs were completed for each extract. The fluorescence measurements were normalized to the curve of the blank (no antioxidant). From the normalized curves, the area under the fluorescence decay curve (AUC) was calculated. The net AUC corresponding to a sample was calculated as the difference between the sample or Trolox and the blank. The regression equation between the net AUC and the antioxidant concentration was calculated. The ORAC value was estimated by dividing the slope of the latter equation by the slope of the Trolox line obtained for the same assay. The final ORAC values were expressed as the mmol of TE/g of the phenolic extract.

### 2.7. Statistical Analysis

A one-way analysis of variance (ANOVA), followed by Tukey’s post hoc test, was applied to the polyphenol quantification values, and differences were considered significant at *p* < 0.05. Pearson correlation analyses were performed (*p* < 0.05) for all four antioxidant activities, the quantified metabolites, and the total polyphenols. R program version 4.4.2 (2024-10-31 ucrt) was used for statistical analysis.

## 3. Results and Discussion

### 3.1. UPLC-QTOF-ESI MS Analysis

**Figure 1:**
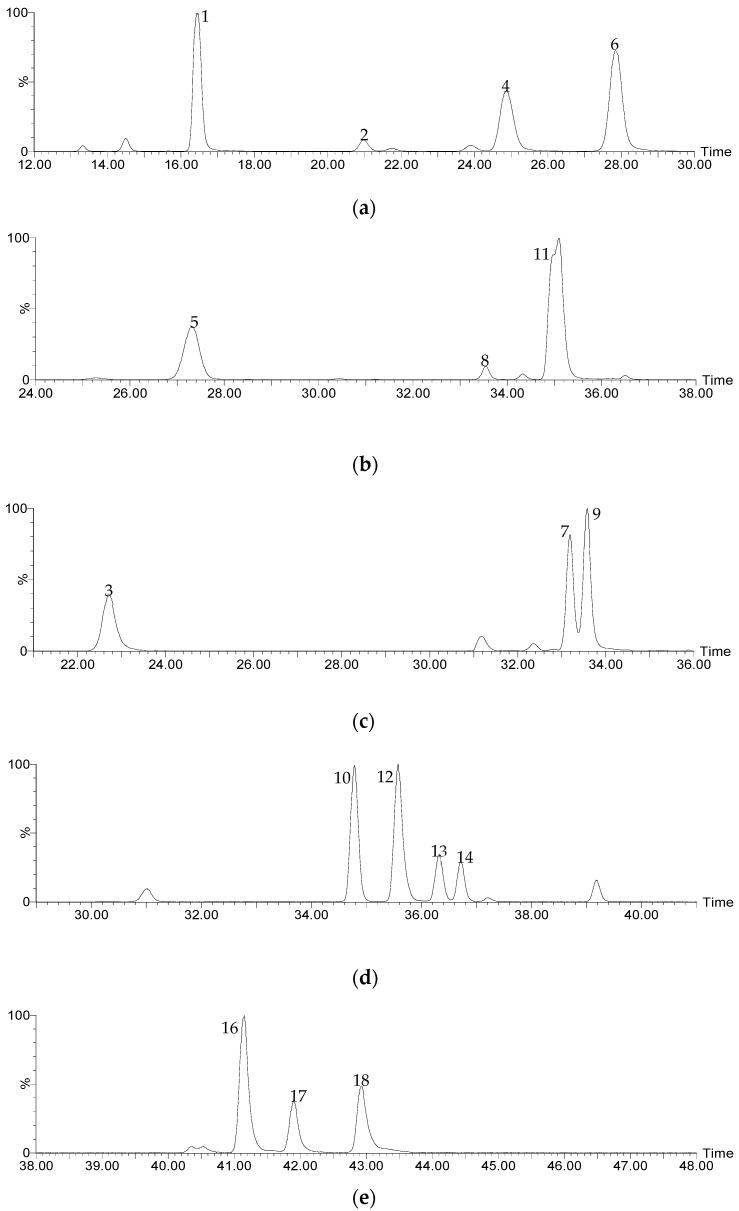
Extracted ion chromatograms (XICs) for the phenolic acids identified through the UPLC-QTOF-ESI MS analysis of spent coffee extracts. (**a**) Isomers of caffeoylquinic acid, (**b**) isomers of feruloylquinic acid, (**c**) isomers of coumaroylquinic acid, (**d**) caffeoylshikimic acid and caffeoylquinic lactone isomers, and (**e**) isomers of dicaffeoylquinic acid.

The results of the UPLC-QTOF-ESI MS analysis, performed as described in Section 2.3, are summarized in Table 1, including the identified compounds, their molecular formulas, the *m*/*z* of the [M-H]^−^ ions, the main ms^2^ fragments, and the respective retention times (Rts) in the chromatogram. Figure 1 shows the ion extraction chromatograms (XICs) for each compound.

As described, 21 different compounds were identified, including CQAs, FQAs, CoQAs, and additional types of polyphenolic acids such as caffeoylshikimic acids and higher molecular mass acids such as dicaffeoylquinic acids. Likewise, three caffeoylquinic lactones and four diterpene atractyligenins were identified.

**Figure 2:**
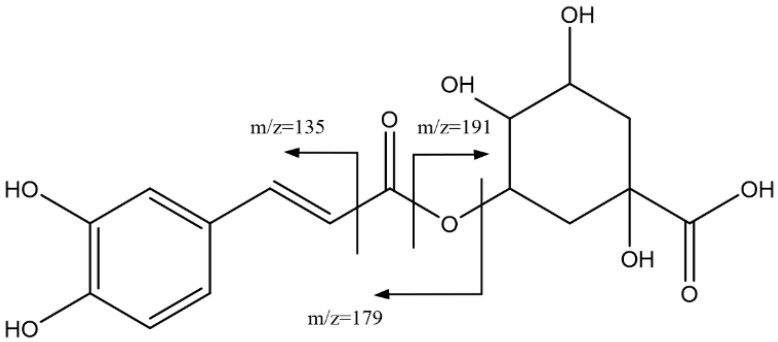
Main fragmentations of caffeoylquinic acid isomers, peaks 1, 2, 4 and 6.

Figure 2 shows the main fragmentations for peaks 1, 2, 4, and 6, with Rts of 16.43 min, 20.96 min, 24.86 min, and 27.84 min, respectively, which were identified as isomers of caffeoylquinic acid which have previously been reported in the literature [37]. Indeed, these peaks had a pseudomolecular ion [M-H]^−^ = 353.0873 Da, which corresponded to the molecular formula C_16_H_17_O_9_. Also, a peak was observed at 707 Da corresponding to the dimer [2M-H]^−^. The main fragments in these peaks were observed at 191 Da (quinic acid), 179 Da (caffeic acid), and 135 Da, derived from carbonyl α-cleavage [38].

**Figure 3:**
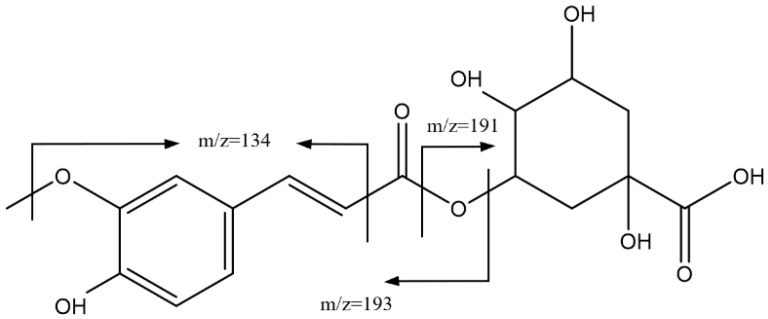
Main fragmentations of isomers of feruloylquinic acid 5, 8, and 11.

Figure 3 shows the main fragmentations for peaks 5, 8, and 11, with Rts of 27.31 min, 33.54 min, and 35.08 min, respectively, with the ion [M-H]^−^ = 367.1026 Da (C_17_H_19_O_9_) corresponding to feruloylquinic acid isomers. This was confirmed by the presence of the adduct [2M-H]^−^ at 735 Da, as well as by the fragmentation pattern, which included peaks at 193 Da (ferulic acid), 191 Da (quinic acid), 173 Da for the loss of H_2_O from quinic acid, and 134 Da for the loss of CH_3_ in conjunction with carbonyl α-cleavage [38].

**Figure 4:**
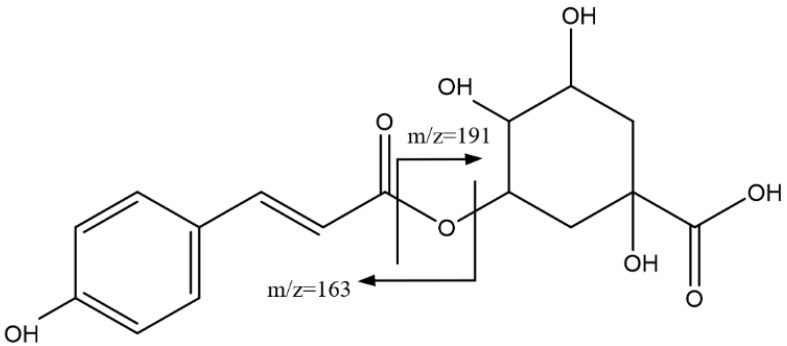
Main fragmentations of isomers of coumaroylquinic acid 3, 7, and 9.

Peaks 3, 7, and 9 (Figure 4), with Rts of 22.69 min, 33.17 min, and 33.61 min, respectively, and [M-H]^−^ = 337.0921 Da (C_16_H_17_O_8_), were assigned as isomers of coumaroylquinic acid. Accordingly, fragments at 191 Da (quinic acid) and 163 Da (coumaric acid) were observed [38].

**Figure 5:**
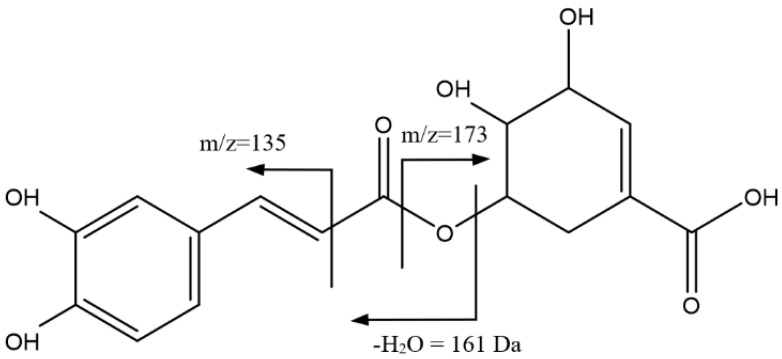
Main fragmentations of an isomer of caffeoylshikimic acid, 10.

Figure 5 shows the main fragmentations of peak 10 (34.78 min) with a mass of [M-H]^−^ = 335.0767 Da (C_16_H_15_O_8_). This peak was identified as a caffeoylshikimic acid isomer, with fragments at 173 Da due to shikimic acid, 161 Da from caffeic acid with the loss of an additional H_2_O molecule, and 135 Da from carbonyl α-cleavage [39,40].

**Figure 6:**
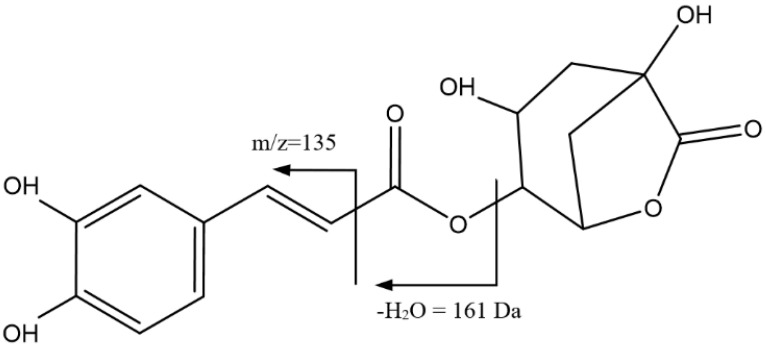
Main fragmentations of caffeoylquinic lactone isomers 12, 13, and 14.

Figure 6 shows the main fragmentations of peaks 12, 13, and 14, for which a mass of [M-H]^−^ = 335.0765 Da (C_16_H_15_O_8_) was observed, with Rts of 35.57, 36.30, and 36.71 min, respectively. These peaks were identified as isomers of caffeoylquinic lactones, with a fragment at 135 Da due to carbonyl α-cleavage and at 161 Da from caffeic acid with the loss of an additional H_2_O molecule, which in turn generated the fragment at 133 Da due to the additional loss of CO, in line with the literature [39].

**Figure 7:**
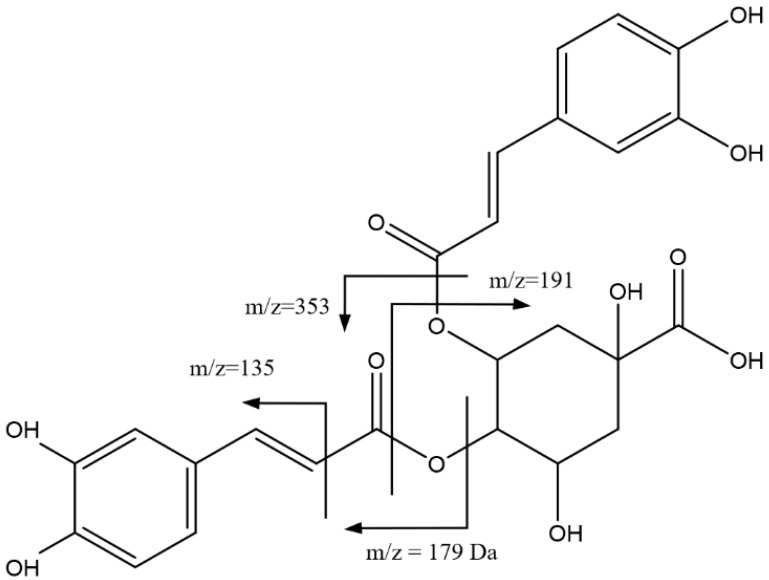
Main fragmentations of isomers of dicaffeoylquinic acid 15, 16, and 17.

For peaks 16, 17, and 18, with Rts of 41.15 min, 41.89 min, and 42.91 min, respectively, there was an observed ion [M-H]^−^ = 515.1187 Da (C_25_H_23_O_12_), corresponding to dicaffeoylquinic acid isomers (Figure 7). The main fragments were observed at 353 Da due to the loss of one of the caffeoyl moieties, at 191 Da corresponding to quinic acid, at 179 Da due to the caffeic acid fragment, and at 135 Da from carbonyl α-cleavage [38].

**Figure 8:**
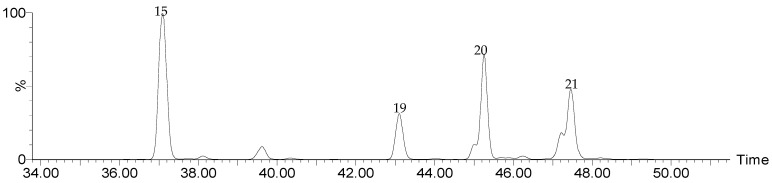
Ion extraction chromatograms (XICs) for the actratylogenins 15, 19, 20, and 21 identified through the UPLC-QTOF-ESI MS analysis of spent coffee extracts.

Figure 8 below shows the different ion extraction chromatograms (XICs) for the atractyligenin-type diterpenes.

**Figure 9:**
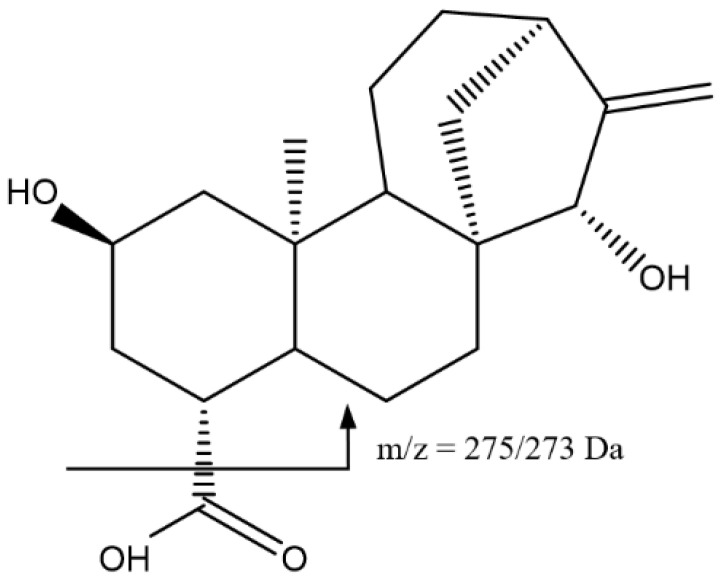
Main fragmentations for the aglycone atractyligenin, 19.

Peak 19 at 43.10 min had an ion [M-H]^−^ = 319.1903 Da, which corresponded to the formula C_19_H_27_O_4_ (Figure 9). This peak was assigned to the atractyligenin aglycone, with fragments at 275 Da and 273 Da due to the loss of the acid group in the form of CO_2_ and HCO_2_H, respectively [41,42].

**Figure 10:**
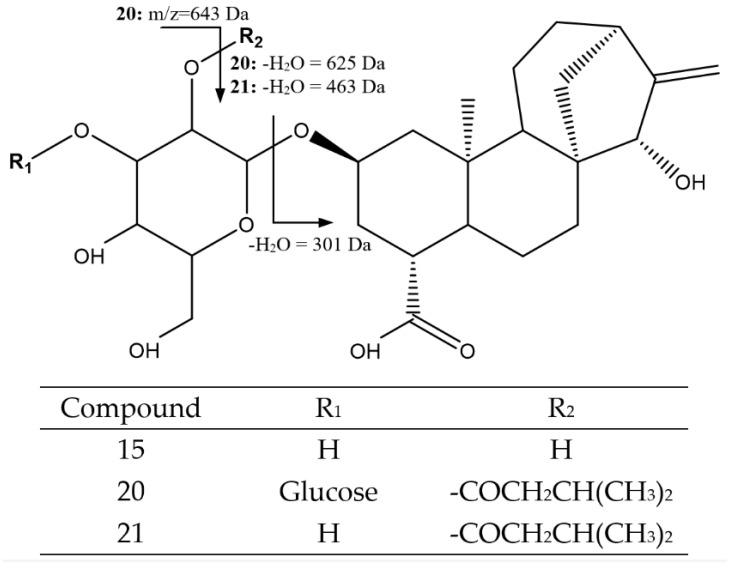
Structure and main fragmentation for attractyligenin glycosides 15, 20, and 21.

Peak 15, at 37.08 min, had an [M-H]^−^ ion at 481.2448 Da (C_25_H_37_O_9_), corresponding to atractyligenin-2-*O*-glucoside (Figure 10), with the main fragment at 301 Da due to the loss of the glycoside along with an additional H_2_O molecule [41,43]. Peak 20 (45.24 min, [M-H]^−^ = 727.3541 Da, C_36_H_55_O_15_) was assigned to atractyligenin-3′-*O*-glucopyranosyl-2′-isovaleryl-2-*O*-glucopyranoside, with fragments at 643 Da due to the loss of the isovaleryl group and at 625 Da from the additional loss of a molecule of H_2_O [42,43].

Finally, peak 21 at 47.46 min was assigned to atractyligenin-2′-*O*-isovaleryl-2-*O*-glucopyranoside, with [M-H]^−^ = 565.3007 Da (C_30_H_45_O_10_) and characteristic fragments at 463 Da due to the loss of isovaleryl and an additional H_2_O molecule and at 301 Da from the cleavage of the glucoside along with a molecule of H_2_O [42].

The presence of these metabolites makes SCGs a valuable material for food applications with potential health advantages. For instance, lipid-lowering and glucose-modulating effects have been reported for the CGAs identified in this study [20,21], indicating their potential application in functional foods targeting metabolic health, such as weight management or diabetes-friendly products. Additionally, their antihypertensive and anticancer properties further broaden their potential in designing therapeutic foods or supplements [22]. In turn, the atractyligenins identified in this work have been reported to offer novel opportunities for creating functional ingredients that support liver health, glucose regulation, and skin protection [28,29,30,31], aligning with consumer trends for holistic wellness solutions. These bioactive compounds exemplify the potential of spent coffee-derived ingredients in addressing health concerns through functional and therapeutic food innovations.

### 3.2. Total Polyphenols

The Folin–Ciocalteu (FC) method is widely used to quantify the total polyphenols in a sample. The method consists of reducing a complex of phosphomolybdic acid and phosphotungstic acid through the donation of electrons made by phenolic groups. Due to the mechanism by which the reaction occurs, the FC method is a single electron transfer (SET) method, which is why it is also considered a means of analyzing the antioxidant capacity [44].

Section 2.4 describes the procedure used in this study, and Table 2 summarizes the values obtained.

The above results showed that the polyphenol content of the GR and PRC samples was significantly lower than that of the EXP sample. The EXP sample had a similar value to that found by Wu et al. [45], who reported 9.44 mg GAE/g dry weight (DW), and a higher value than those reported by Mussatto et al. [46], which ranged from 6.0 to 7.4 mg GAE/g DW. Other references using spent coffee beans’ hydroalcoholic extracts indicated slightly higher values of the polyphenolic content [37,47,48].

### 3.3. UPLC-DAD Analyses

A UPLD-DAD analysis was conducted as described in Section 2.5. The results for the quantification of caffeine in the spent coffee samples are presented in Table 3.

The caffeine content of the EXP sample was higher than that of the GR and PRC samples. In fact, the EXP content showed a value within the range of 2.5–7.9 mg/g DW reported by Cruz et al. [49] for spent espresso coffee. Other previous results obtained for extracts in aqueous ethanol showed a wider range of 0.3–20 mg/g DW [48], which include the values obtained in the present work.

A UPLC-DAD analysis was also carried out to quantify the phenolic compounds, as described in Section 2.5. Table 4 shows the results obtained for the spent coffee samples EXP, GR, and PRC.

As shown in Table 4, the fine-grind sample (EXP) had the highest content of CQAs, (peaks 1, 4, and 6) corresponding to 87% of the total quantifiable phenolic acids, while FQAs (peaks 5, 8, and 11) accounted for 13% of the total. This result differs from that of the medium-grind sample (GR), which showed a similar content of both types of acids, with 56% CQAs and 44% FQAs. Meanwhile, CQAs and FQAs accounted for 36% and 64%, respectively, in the coarse-grind sample (PRC).

Compared with the literature, the total concentration of phenolics obtained in the GR and PRC samples was within the range of results previously reported for spent coffee, ranging between 0.21 and 0.77 mg/g DW [46,49], while the EXP sample had significantly higher values than these reports. Angeloni et al. [40] reported one sample of Costa Rican spent coffee with a phenolic content of 1.64 mg/g DW in a hydroalcoholic extract, which is lower than the value obtained in our study for the EXP sample. Another study reported a methanolic extract with a phenolic content of 0.58 mg/g DW [50], which is similar to the values we found for the GR and PRC samples.

### 3.4. Antioxidant Activity Determination

Table 5 summarizes the results obtained from analyzing the antioxidant activities of the different SCG extracts.

The above results are discussed for each antioxidant methodology in the following sections. Table 6 summarizes the data in relation to the dry mass of the spent coffee samples.

#### 3.4.1. DPPH

DPPH analysis uses the free radical 2,2-diphenyl-1-picrylhydrazyl. In this test, the intensity of the purple color produced by the DPPH* radical, which decreases due to the action of antioxidant compounds, is quantified using spectrophotometry. The mechanism of the reaction is mixed, being carried out by both electron transfer (SET) and hydrogen atom transfer (HAT) [44]. As previously mentioned, the results are expressed as the IC_50_ value, which indicates the sample concentration that produces a 50% inhibition of the DPPH* free radical.

The results showed a lower IC_50_ value for the fine-grind EXP sample compared to the GR and PRC samples, again indicating an antioxidant value that surpasses those of previous reports in the literature for spent espresso coffee, which range between 0.4 and 1.4 g DW/L [37,45]. The present findings are consistent with the literature for both coffee and spent coffee extracts, with a higher polyphenol content aligning with a better result regarding the antioxidant activity [46,51].

#### 3.4.2. PFRAP

The PFRAP method is an analysis that employs the SET mechanism and is based on the reduction of an iron (III) complex to iron (II) to form Prussian blue, which is observed through the formation of an intense blue color measured using spectrophotometry. Unlike the other antioxidant analyses carried out, PFRAP does not involve radicals, which is why it constitutes a complementary technique in the evaluation of antioxidant activity [44].

As with the previous tests, the PFRAP results revealed the EXP sample as having the highest antioxidant activity compared to the GR and PRC samples. The EXP sample also showed similar values to those reported in the literature, which range from 0.20 to 0.22 mmol FeSO_4_/g DW [46] when using the similar FRAP (ferric-reducing antioxidant power) technique.

#### 3.4.3. ORAC

The ORAC assay is performed by measuring the kinetics of fluorescence at a known concentration of sodium fluoresceinate. The radicals are peroxide groups emitted by the AAPH reagent, which react with fluorescein, and then the loss of fluorescence is recorded. This loss is inhibited in the presence of an antioxidant compound, which reacts through a HAT mechanism with the peroxyl radicals, allowing the fluorescence signal to last longer [52].

In this analysis, the GR and PRC samples’ results were similar, with the GR being slightly higher in its antioxidant capacity, while the EXP sample gave a significantly higher value, following a similar trend to the antioxidant activity evaluations described above. In the literature, a value of 735–823 μmol Trolox/g DW was reported for coffee fruits [53], indicating that the EXP spent coffee sample retained nearly half of its antioxidant activity in the ORAC assay.

#### 3.4.4. TEAC

In TEAC analysis, the intensity of the coloration of the blueish-green ABTS*^+^ radical is measured using spectrophotometry. This radical is formed by the reaction between an ABTS solution and an oxidant such as potassium persulfate. In the presence of antioxidants, these react with ABTS*^+^ radicals and cause the discoloration of the solution, which is then compared against a curve prepared using Trolox as the standard [54].

In this analysis, the GR and PRC samples showed similar results to those obtained by Panusa et al. [43] for coffee silverskin, with values ranging from 6.14 to 15.63 mg Trolox/g D. The fine-grind EXP sample in our study showed higher values, which aligned with the 58.62 mg Trolox/g DW reported by Panusa et al. for a green coffee bean sample. Again, these results show that the EXP spent coffee sample retained almost half of its antioxidant activity.

**Figure 11:**
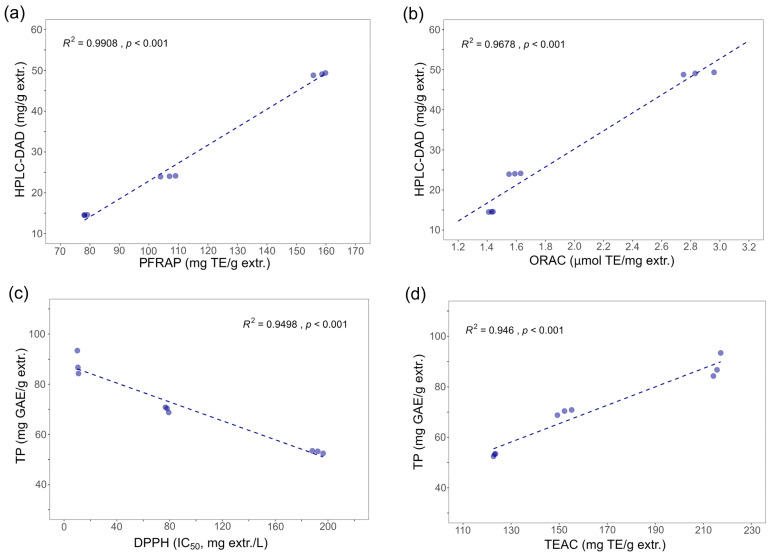
Correlations between phenolics content and antioxidant activity evaluations: (**a**) UPLC-DAD quantification and PFRAP antioxidant values, (**b**) UPLC-DAD quantification and ORAC antioxidant values, (**c**) total polyphenols (TP) and DPPH antioxidant values, and (**d**) TP and TEAC antioxidant values.

Finally, a Pearson correlation study was performed to evaluate the relationship between the polyphenolic content and the antioxidant activity evaluations. The results are summarized in Table 7 and Figure 11.

As can be observed in Table 7, significant positive correlation values (*p* < 0.05) were obtained for the relationship between the antioxidant activities as indicated by the ORAC, TEAC, and PFRAP and the total polyphenols evaluation (using the FC method) and the quantification of polyphenolic acids using HPLC-DAD. This finding aligns with previous studies showing that the phenolic compounds content correlates with the antioxidant activity [55,56].

Likewise, significant negative correlation values (*p* < 0.05) were found between the results of the DPPH assay and the other antioxidant activities, as well as between the TP values and the polyphenolic acid quantification results. This negative correlation is due to the DPPH method’s measurement of an inhibition value, which means a lower numerical result implies a better antioxidant capacity, thus cohering with a higher amount of phenolics [57].

These promising results on the phenolic content and antioxidant potential align with previous reports on other natural Costan Rican products [58] that call for further phytochemical and bioactivity studies of these types of extracts. CGAs and related compounds in coffee hold significant potential for innovative food applications due to their diverse bioactive properties. As natural antioxidants, CGAs can enhance the shelf life and stability of food products by reducing oxidative damage, while their anti-inflammatory effects offer functional health benefits [15,16,17,18,19], appealing to the growing demand for nutraceuticals. FQAs and CoQAs, with their potent antioxidative and anti-inflammatory capacities [23,24,25,26,27], could enhance the nutritional profile of beverages, snacks, or dietary supplements.

However, while in vitro antioxidant assays provide valuable insights, they do not fully represent the bioavailability, metabolism, and physiological effects of the compounds in vivo. Therefore, other studies are needed to capture the full spectrum of bioactive compounds present in SCGs. Moreover, the nature of the spent coffee, for instance the variability in the polyphenol content based on the coffee type, grind size, and processing conditions, may impact the consistency in food formulations. In addition, large-scale extraction and processing methods for SCG utilization must address compliance with food safety regulations, the cost effectiveness, and infrastructure limitations [59]. Therefore, despite the importance of the present results, further work is crucial to fully assess the potential and viability of these SCG extracts for commercial applications.

## 4. Conclusions

This work constitutes the first comprehensive report on extracts of spent coffee grounds from Costa Rica, with a total of twenty-one compounds tentatively identified using UPLC-QTOF-ESI MS, including fourteen phenolic acids, three caffeoylquinic lactones, and four atractyligenin diterpenes. The sample with the highest value for the total polyphenols, the fine-grind spent coffee, also showed a higher UPLC-DAD phenolic acid content and better performance in the DPPH, PFRAP, TEAC, and ORAC antioxidant evaluations compared to the medium-grind sample, the coarse-grind sample, and even previous reports in the literature. Even though these are promising results, further studies are needed to determine the potential of this waste material and its antioxidant properties.

## Figures and Tables

**Table 1:** Main compounds in spent coffee extracts identified through UPLC-Q-TOF analysis.

No.	Compound	Rt (min)	[M-H]^−^	Molecular Formula	ms2 Fragments
1	Caffeolylquinic acid (isomer 1 of 4)	16.43	353.0876	C_16_H_17_O_9_	201, 191, 179, 135
2	Caffeoylquinic acid (isomer 2 of 4)	20.96	353.0869	C_16_H_17_O_9_	191
3	Coumaroylquinic acid (isomer 1 of 3)	22.69	337.0921	C_16_H_17_O_8_	163
4	Caffeoylquinic acid (isomer 3 of 4)	24.86	353.0872	C_16_H_17_O_9_	191
5	Feruloylquinic acid (isomer 1 of 3)	27.31	367.1026	C_17_H_19_O_9_	193, 173, 134
6	Caffeoylquinic acid (isomer 4 of 4)	27.84	353.0869	C_16_H_17_O_9_	201, 191, 179, 173, 135
7	Coumaroylquinic acid (isomer 2 of 3)	33.17	337.0923	C_16_H_17_O_8_	191, 163
8	Feruloylquinic acid (isomer 2 of 3)	33.54	367.1025	C_17_H_19_O_9_	191, 173, 134
9	Coumaroylquinic acid (isomer 2 of 3)	33.61	337.0921	C_16_H_17_O_8_	191, 173, 163
10	Caffeoylshikimic acid	34.78	335.0767	C_16_H_15_O_8_	173, 161, 135,133
11	Feruloylquinic acid (isomer 3 of 3)	35.08	367.1026	C_17_H_19_O_9_	191, 173, 134
12	Caffeoylquinic lactone (isomer 1 of 3)	35.57	335.0766	C_16_H_15_O_8_	161, 135, 133
13	Caffeoylquinic lactone (isomer 2 of 3)	36.30	335.0763	C_16_H_15_O_8_	161, 135, 133
14	Caffeoylquinic lactone (isomer 3 of 3)	36.71	335.0768	C_16_H_15_O_8_	161, 133
15	Atractyligenin-2-O-glucopyranoside	37.08	481.2448	C_25_H_37_O_9_	301, 119
16	Dicaffeoylquinic acid (isomer 1 of 3)	41.15	515.1190	C_25_H_23_O_12_	353, 335, 191, 179, 173, 161, 135
17	Dicaffeoylquinic acid (isomer 2 of 3)	41.89	515.1187	C_25_H_23_O_12_	375, 353, 191, 179, 135
18	Dicaffeoylquinic acid (isomer 3 of 3)	42.91	515.1185	C_25_H_23_O_12_	353, 191, 179, 173, 135
19	Atractyligenin	43.10	319.1903	C_19_H_27_O_4_	275, 273
20	Atractyligenin-3′-O-glucopyranosyl-2′-isovaleryl-2-O-glucopiranoside	45.24	727.3541	C_36_H_55_O_15_	643, 625
21	Atractyligenin-2′-isovaleryl-2-O-glucopiranoside	47.46	565.3007	C_30_H_45_O_10_	473, 3

**Table 2:** Total polyphenols of spent coffee samples EXP, GR, and PRC.

Sample	Total Polyphenols (mg GAE/g Extract) ^1,2^	Total Polyphenols (mg GAE/g Dry Weight) ^1,2^
EXP	88.1 ^a^ ± 3.8	9.59 ^a^ ± 0.42
GR	70.0 ^b^ ± 0.9	2.13 ^b^ ± 0.03
PRC	53.1 ^c^ ± 0.5	1.70 ^c^ ± 0.01

^1^ Values are expressed as average ± standard deviation. ^2^ Different superscript letters in same column indicate significant differences (*p* < 0.05) using one-way analysis of variance (ANOVA) with post hoc Tukey test as statistical analysis test.

**Table 3:** Caffeine quantification using UPLC-DAD on samples of spent coffee.

Sample	Concentration (mg/g Extract) ^1,2^	Concentration (mg/g DW) ^1,2^
EXP	31.435 ^a^ ± 0.012	3.4201 ^a^ ± 0.0014
GR	18.040 ^b^ ± 0.008	0.5496 ^b^ ±0.0002
PRC	54.020 ^c^ ± 0.065	1.7294 ^c^ ± 0.0021

^1^ Values are expressed as average ± standard deviation. ^2^ Different superscript letters in same column indicate significant differences (*p* < 0.05) using one-way analysis of variance (ANOVA) with post hoc Tukey test as statistical analysis test.

**Table 4:** Quantification of caffeoylquinic and feruloylquinic acids in spent coffee samples using UPLC-DAD.

Compound No.	Concentration (mg/g Extract) ^1,2^			Concentration (mg/g DW) ^1,2^		
	EXP	GR	PRC	EXP	GR	PRC
**1**	12.026 ^a^ ± 0.026	5.495 ^a^ ± 0.036	1.854 ^a^ ± 0.060	1.3085 ^a^ ± 0.0026	0.1674 ^a^ ± 0.0014	0.0593 ^a^ ± 0.0024
**4**	18.101 ^b^ ± 0.13	3.800 ^b^ ± 0.033	1.861 ^a^ ± 0.016	1.9695 ^b^ ± 0.0141	0.1158 ^b^ ± 0.0012	0.0596 ^a^ ± 0.0006
**5**	1.617 ^c^ ± 0.003	4.098 ^c^ ± 0.001	3.742 ^b^ ± 0.043	0.1760 ^c^ ± 0.0003	0.1249 ^c^ ± 0.0001	0.1198 ^b^ ± 0.0017
**6**	12.768 ^d^ ± 0.070	4.127 ^c^ ± 0.010	1.554 ^c^ ± 0.013	1.3893 ^d^ ± 0.0070	0.1257 ^c^ ± 0.0004	0.0497 ^c^ ± 0.0005
**8**	2.697 ^e^ ± 0.022	3.001 ^d^ ± 0.017	2.628 ^d^ ± 0.042	0.2934 ^e^ ± 0.0022	0.0914 ^d^ ± 0.0006	0.0842 ^d^ ± 0.0016
**11**	1.859 ^f^ ± 0.039	3.524 ^e^ ± 0.021	2.897 ^e^ ± 0.068	0.2023 ^f^ ± 0.0039	0.1074 ^e^ ± 0.0008	0.0927 ^e^ ± 0.0008
**Total**	49.068	24.045	14.536	5.3390	0.7326	0.4653

^1^ Values are expressed as average ± standard deviation. ^2^ Different superscript letters in same column indicate significant differences (*p* < 0.05) using one-way analysis of variance (ANOVA) with post hoc Tukey test as statistical analysis test.

**Table 5:** Antioxidant activities measured for extracts of spent coffee samples.

Sample	DPPH	PFRAP		ORAC	TEAC
	IC_50_ (mg Extract/L) ^1,2^	mg Trolox/g Extract ^1,2^	mmol FeSO_4_/g Extract ^1,2^	μmol Trolox/mg Extract ^1,2^	mg Trolox/g Extract ^1,2^
EXP	10.5 ^a^ ± 0.4	158.1 ^a^ ± 1.7	1.273 ^a^ ± 0.020	2.847 ^a^ ± 0.087	215.7 ^a^ ± 1.2
GR	78.1 ^b^ ± 1.0	106.6 ^b^ ± 2.1	0.921 ^b^ ± 0.025	1.590 ^b^ ± 0.033	152.2 ^b^ ± 2.4
PRC	192.2 ^c^± 3.3	78.5 ^c^ ± 0.5	0.672 ^c^ ± 0.006	1.427 ^b^ ± 0.012	123.1 ^c^ ± 0.4

^1^ Values are expressed as average ± standard deviation. ^2^ Different superscript letters in same column indicate significant differences (*p* < 0.05) using one-way analysis of variance (ANOVA) with post hoc Tukey test as statistical analysis test.

**Table 6:** Antioxidant activities measured for samples of spent coffee, expressed per gram of dry weight.

Sample	DPPH	PFRAP		ORAC	TEAC
	IC_50_ (g DW/L) ^1,2^	mg Trolox/g DW ^1,2^	mmol FeSO_4_/g DW ^1,2^	μmol Trolox/g DW ^1,2^	mg Trolox/g DW ^1,2^
EXP	0.096 ^a^ ± 0.003	17.20 ^a^ ± 0.19	0.1385 ^a^ ± 0.0022	309.7 ^a^ ± 9.4	23.47 ^a^ ± 0.14
GR	2.562 ^b^ ± 0.034	3.25 ^b^ ± 0.06	0.0281 ^b^ ± 0.0008	48.4 ^b^ ± 1.0	4.64 ^b^ ± 0.07
PRC	6.005 ^c^ ± 0.104	2.51 ^c^ ± 0.02	0.0215 ^c^ ± 0.0002	45.7 ^b^ ± 0.4	3.94 ^c^ ± 0.01

^1^ Values are expressed as average ± standard deviation. ^2^ Different superscript letters in same column indicate significant differences (*p* < 0.05) using one-way analysis of variance (ANOVA) with post hoc Tukey test as statistical analysis test.

**Table 7:** Pearson correlation values (*p* < 0.05) obtained for the quantification of polyphenolic acids and antioxidant activity evaluations.

	HPLC-DAD	TP	DPPH	PFRAP	ORAC
TP	0.962				
DPPH	−0.920	−0.975			
PFRAP	0.995	0.981	−0.950		
ORAC	0.984	0.920	−0.842	0.967	
TEAC	0.998	0.973	−0.936	0.999	0.977

## Data Availability

The original contributions presented in this study are included in the article. Further inquiries can be directed to the corresponding author.
